# Aging effects on the mechanical energy transfer through the lower extremity joints during the swing phase of level walking

**DOI:** 10.1038/s41598-019-45267-z

**Published:** 2019-07-02

**Authors:** Hung-Bin Chen, Liang-Wey Chang, Chih-Hsiu Cheng

**Affiliations:** 1 Institute of Biomedical Engineering, College of Medicine and College of Engineering, National Taiwan University, Taipei, Taiwan R.O.C.; 2 School of Physical Therapy and Graduate Institute of Rehabilitation Science, College of Medicine, Chang Gung University, Taoyuan, Taiwan R.O.C.; 3 Bone and Joint Research Center, Chang Gung Memorial Hospital, Linkou, Taoyuan, Taiwan R.O.C.

**Keywords:** Orthopaedics, Rehabilitation

## Abstract

Age-related changes of gait performance have been evidenced by the altered kinetic coordination of the lower extremity. However, a systematic approach to explore the gait control in terms of the mechanical energy transfer across multiple segments throughout the gait phases is still not well documented. Ten healthy elderly and ten young adults were asked to walk along a 10-meter walkway at the self-selected and fast walking speeds. The visualized energy flow model of the swing leg was established and the factor analysis was then applied to extract the high-dimensional energy flow characteristics of the swing leg. The results showed that the young adults have similar energy flow characteristics of the swing leg for both fast and self-selected walking speeds, while the elderly showed an opposite energy flow pattern especially at the fast walking speed. The hip power and the knee power were also found to mainly correspond to the swing acceleration and deceleration, respectively. This study demonstrated a valuable tool to explore the change of the gait characteristics in the elderly and could help to facilitate the understanding of the neuromuscular adaptation due to aging.

## Introduction

Age-related loss of strength, balance control, and cardiorespiratory function can limit locomotion ability and result in slower walking speed, shorter step length, and deterioration of balance control^1,2^. Change of gait performance could be driven by the altered neuromuscular control since the physical degenerations need to be compensated by adjusting the kinematic and kinetic coordination of the body. Previous studies reported that the healthy elderly showed reduced ankle plantarflexion angle/moment/power, reduced knee flexion angle, reduced knee extension moment/power, reduced hip extension angle, and increased hip flexion/extension moment/power^3–6^. These evidences suggested that aging could lead to neuromuscular adaption of the lower extremity that occurs in multiple joints rather than a single one.

During the swing phase of the gait cycle, the elderly has been reported to exhibit greater toe clearance variability than the young adults^7,8^, while the variability should be minimized to avoid the occurrence of toe-ground contact events. Tripping occurred during the swing phase of walking had been reported to be responsible for 53% of falls in elderly^9^. Previous studies also reported that, while making a rapid voluntary forward step for the advancement of the lower extremity, balance-impaired older adults demonstrate smaller step length, slower step reaction time, and longer step time than balance-unimpaired older adults, and these measures are closely related to fall risks^10–12^. These findings highlight the importance to identify the age-related changes of the movement strategy during the swing phase that may predispose the elderly to slipping or tripping.

Energy flow analysis had been utilized to investigate the mechanical powers across multiple segments and joints^13,14^. A previous study found an energy flow disruption prior to push-off in elderly that is an altered hip mechanics to compensate the ankle plantarflexor weakness during a fast walking^15^. Another study also found that the elderly with orthopedic problems spend more hip mechanical energy to compensate for the weak ankle plantarflexor^11^. However, previous studies mostly focused only on specific gait events, e.g. at the instance of the foot push-off, which may not be able to identify the comprehensive neuromuscular adaptation of a gait cycle. It can be very challenging to analyze the energy flow characteristics of different gait phases due to the high-dimensionality, temporal-dependence, and high variability of the dataset. To our knowledge, there is no systematic approach to characterize the energy flow pattern during the entire phases, either the stance phase or the swing phase, of the gait.

The purposes of this study were to identify the important factors that control the swing leg and to compare the energy flow characteristics of the swing phase during level walking in young adults and the elderly. Factor analysis would be utilized to extract the characteristics of the energy flows from a high-dimensional dataset. Our research hypothesis was that the elderly presents distinct energy flow characteristics that unveil the altered coordination among the segments of the lower extremity during the swing phase. Our work would help to facilitate the understanding of the neuromuscular adaptation due to aging, which can potentially contribute to the fall prevention, orthopedic treatments and rehabilitation interventions for elderly.

## Materials and Methods

### Subjects

Ten healthy elderly (mean age: 68.1 ± 6.4 years; 5 females and 5 males) who could walk without any assistance or aids, and ten healthy young adults (mean age: 25.1 ± 1.6 years; 2 females and 8 males) participated in this study. Exclusion criteria included the inability to follow instructions, cardiopulmonary dysfunctions, joint replacements in the lower extremities, arthritis, diabetes, vestibular deficits, or any type of neuromusculoskeletal problems that could interfere with the gait pattern. All the participants were right foot dominant, defined as the preferred leg for kicking a ball. The experimental protocol was approved by and performed in accordance with the relevant guidelines and regulations of the Research Ethics Committee of National Taiwan University Hospital (No. 201112121RIC). All subjects had provided their signed informed consents before participating in the study.

### Instrumentation

Eleven segmental landmarks of the subject’s body were tracked at 50 Hz by the optoelectric motion capture system (Optotrak Certus, Northern Digital Inc., Waterloo, Canada) for the three-dimensional gait analysis. Those segmental landmarks were defined by the modified Helen Hayes marker set as follows^16^: mid-point of right and left posterior-superior-iliac-spine, bilateral anterior-superior-iliac-spine, right greater trochanter, right lateral femoral condyle, right medial femoral condyle, right fibular head, right lateral malleolus, right medial malleolus, right heel, and right second metatarsal head.

### Experimental protocol

All subjects walked with shoes along a 10-meter walkway at the self-selected speed and the fast speed respectively. The instruction for the fast speed was to ask the subject simulating a functional task, i.e. crossing a street as fast as possible when the green light is about to turn red in 10 seconds. Each subject was allowed to practice and completed at least three successful gait trails. A trial was considered “successful” when constant walking speed was obtained during gait. Trajectories of all markers attached to the subject during the 10-meter walking were recorded and then filtered with a fourth-order, bidirectional Butterworth low-pass filter with a cutoff frequency of 10 Hz. The kinematic data of swing phase were normalized to the duration of the swing phase (yielding the relative time profiles between 0% and 100%) and were averaged over the three recorded trials. Those normalized kinematic data would further be used to calculate the energy flow of the lower extremity.

### Energy flow model

There are four core elements defining the energy flow of the human body^14^, including the segmental proximal flow (${\dot{{\rm{E}}}}_{{\rm{p}}}$), segmental distal flow (${\dot{{\rm{E}}}}_{{\rm{d}}}$), segmental energy change rate (${\dot{{\rm{E}}}}_{{\rm{s}}}$), and joint power (P). The segmental proximal/distal flow indicates the energy entering or leaving a segment at its proximal or distal part, the segmental energy change rate describes the energy flow within a segment, and the joint power describes the energy flow across a joint. The segmental proximal/distal flow was calculated as follows:

$${\dot{{\rm{E}}}}_{{\rm{p}}}\,(\mathrm{or}\,{\dot{{\rm{E}}}}_{{\rm{d}}})={\rm{F}}\cdot {\rm{v}}+{\rm{M}}\cdot {{\rm{\omega }}}_{{\rm{s}}}$$

where v is the linear velocity of a joint center; ω_s_ is the angular velocity of a segment; F and M are the joint force and the joint moment obtained via Newton’s inverse dynamics, respectively. Taking the thigh for example, the positive thigh proximal flow (${\dot{{\rm{E}}}}_{p,{\rm{thigh}}}$) represents there is an energy inflow to the thigh at the hip joint, whereas the negative thigh distal flow (${\dot{{\rm{E}}}}_{d,{\rm{thigh}}}$) represents there is an energy outflow from the thigh to the knee joint.

The segmental energy change rate (${\dot{{\rm{E}}}}_{{\rm{s}}}$) is defined as the rate of mechanical energy change of a segment. Mechanical energy refers to the summation of potential energy and the kinetic energy. According to the rigid body dynamics, the segmental energy change rate also equals to the summation of segmental proximal and distal flows of a segment, i.e. ${\dot{{\rm{E}}}}_{{\rm{s}}}={\dot{{\rm{E}}}}_{{\rm{d}}}+{\dot{{\rm{E}}}}_{{\rm{p}}}$. For example, 50 Watts of ${\dot{{\rm{E}}}}_{p,{\rm{thigh}}}$ and −20 Watts of ${\dot{{\rm{E}}}}_{d,{\rm{thigh}}}$ indicate that ${\dot{{\rm{E}}}}_{s,{\rm{thigh}}}$ increases 30 Watts.

The joint power (P) is commonly calculated by ${\rm{P}}={\rm{M}}\cdot {{\rm{\omega }}}_{{\rm{j}}}$, where ω_j_ is the angular velocity of a joint. The joint power equals to the summation of ${\dot{{\rm{E}}}}_{{\rm{p}}}$ of the distal segment and ${\dot{{\rm{E}}}}_{{\rm{d}}}$ of the proximal segment, e.g. ${{\rm{P}}}_{{\rm{knee}}}={\dot{{\rm{E}}}}_{p,{\rm{shank}}}+{\dot{{\rm{E}}}}_{d,{\rm{thigh}}}$. For example, 50 Watts of ${\dot{{\rm{E}}}}_{p,\mathrm{shank}}$ and −20 Watts of ${\dot{{\rm{E}}}}_{d,\mathrm{thigh}}$ represent that the knee generates 30 Watts of power (${{\rm{P}}}_{{\rm{knee}}}$).

**Figure 1:**
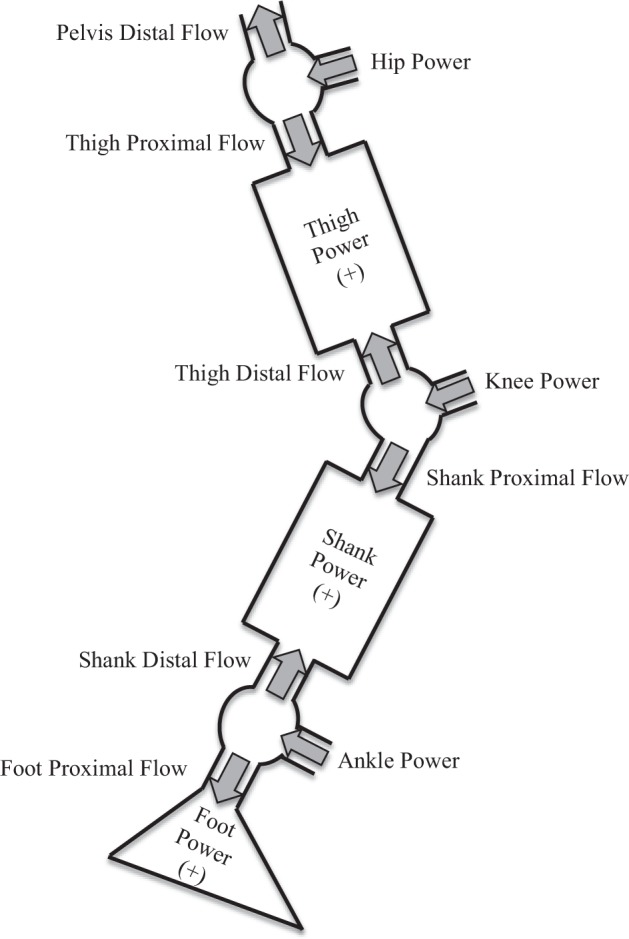
A complete energy flow model of one leg. All energy flows are presented in the positive direction.

A complete energy flow model of one leg was visualized in Fig. 1. Since there is no foot distal flow during the swing phase, i.e. foot does not contact the ground, foot energy change rate is identical to the foot proximal flow. Accordingly, there are eleven energy flow elements in the swing leg model, including the pelvis distal flow, hip power, thigh proximal flow, thigh energy change rate, thigh distal flow, knee power, shank proximal flow, shank energy change rate, shank distal flow, ankle power, and foot proximal flow.

### Data analysis

The energy flow data of all subjects were firstly normalized to the subject’s body mass, and averaged according to the normalized time profiles at 1 percent interval along with the swing duration. A correlation coefficient matrix derived from the normalized averaged data of all the 11 energy flow elements would be produced to evaluate the correlation between each of the energy flow element. The correlation coefficient ranges from 0 to 1 and the higher coefficient indicates the greater correlation. The correlation matrix would be used as inputs for the factor analysis. By rotating the principal components, factor analysis is then utilized to extract the characteristics of a high-dimensional dataset. The extracted 1^st^ and 2^nd^ factors indicate the first two most prominent energy flow patterns of the entire swing phase. For each factor, energy flow elements with significant absolute loadings (>0.8) would further be depicted in the energy flow model while the sign of the loading determined the flow direction of the corresponding energy flow element. Consequently, the energy flow characteristics of the swing leg can be intuitively observed corresponding to each of the extracted factors. The independent t-test was used to compare the walking speeds between the young adults and the elderly.

## Results

For both subject groups, the fast walking speed (1.77 ± 0.15 m/s for the young adults, and 1.56 ± 0.20 m/s for the elderly) was significantly faster (*p* < 0.05) than the self-selected walking speed (1.15 ± 0.24 m/s for the young adults, and 1.11 ± 0.18 m/s for the elderly). The young adults walked significantly faster (*p* = 0.012) than the elderly at the fast walking speed, while not at the self-selected walking speed.

**Figure 2:**
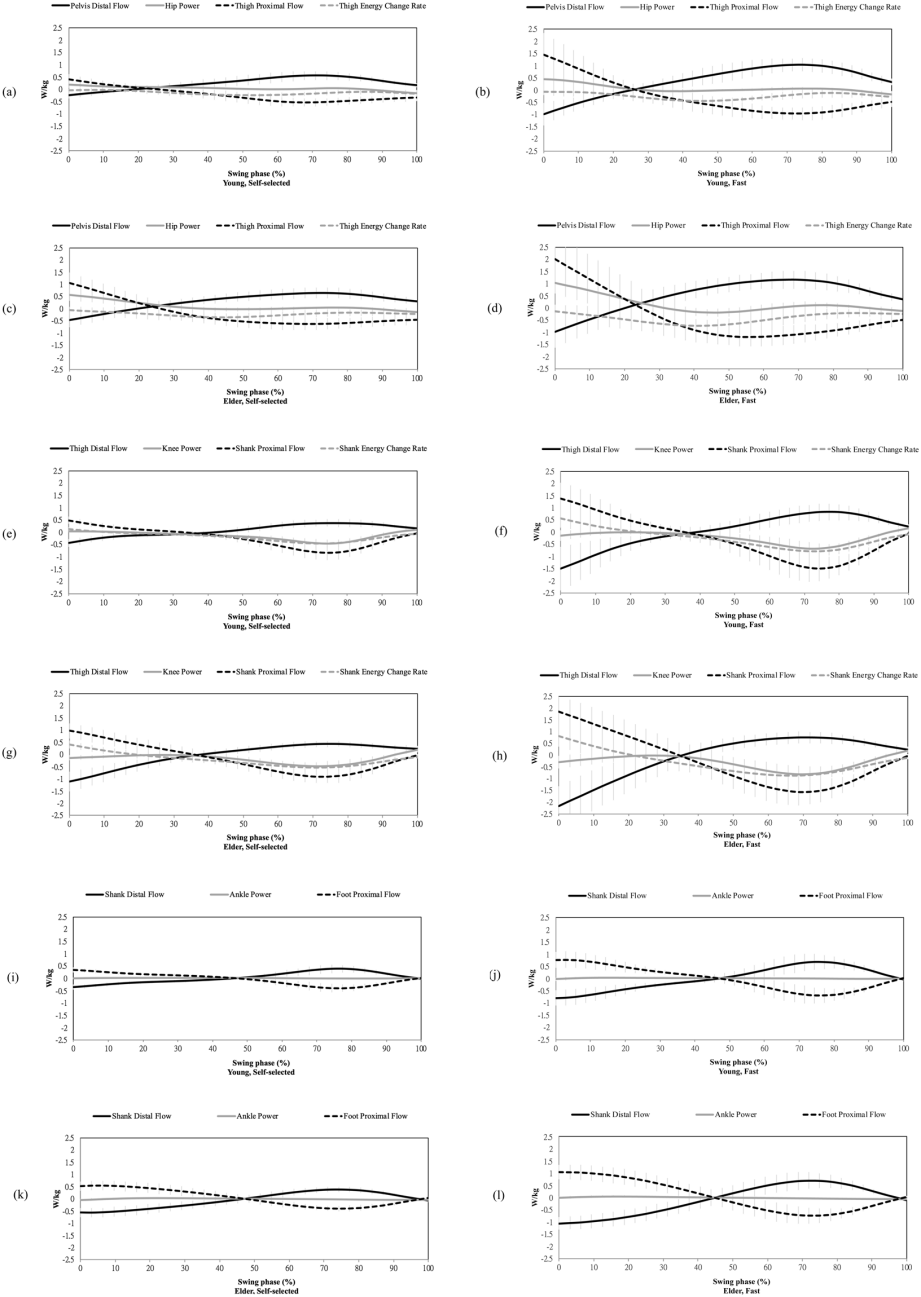
Mean energy flows throughout the whole swing phase in the young adults and the elderly at the self-selected and fast walking speeds. Positive segmental proximal/distal flow represents that the energy flows into the corresponding segment. Positive joint power represents power generation.

Mean profiles of all subjects in terms of the energy flow data of the entire lower extremity during the whole swing phase was shown in Fig. 2, including the energy flow profiles of the thigh in the four conditions as the young adults at the self-selected walking speed (Fig. 2a) and at the fast walking speed (Fig. 2b) as well as the elderly at the self-selected walking speed (Fig. 2c) and at the fast walking speed (Fig. 2d), the energy flow profiles of the shank (Fig. 2e–h), and those of the foot (Fig. 2i–l). In general, the fluctuation trends of the eleven energy flow elements along the swing phase were analogous in all the four conditions. Nevertheless, it was noted that there were great energy fluctuation and great variations among the subjects especially in the elderly at the fast walking speed.

As a representative pattern in the condition of the young adults at the self-selected walking, the early stage of the swing phase showed a negative pelvis distal flow, positive hip power, positive thigh proximal flow, and positive thigh energy change rate (Fig. 2a). In addition, there were negative thigh distal flow, insignificant knee power, positive shank proximal flow, and positive shank energy change rate (Fig. 2e). There were also negative shank distal flow and positive foot proximal flow (Fig. 2i). During the late stage of the swing phase, only the pelvis distal flow, thigh distal flow, and shank distal flow were positive. The rest of the energy flow elements were all negative. The magnitude of ankle power was especially close to zero during the entire swing phase.

The complete correlation matrix of the energy flow elements covaried with each other was shown in Table 1. Notably, the correlation coefficients of ankle power were mostly smaller than 0.1 that could hardly influence the energy flow of the adjacent segments and joints. The ankle power was accordingly excluded in the subsequent analysis. By applying the factor analysis technique, the 1^st^ and 2^nd^ factors were extracted from the correlation matrix since they could explain totally over 90% variance in all conditions (Table 2). Table 3 showed the loadings of all energy flow elements in the extracted 1^st^ and 2^nd^ factors with the signs of the loadings determine the direction of the energy flow (flowing in or flowing out). The significant loadings and the signs of the loadings in the 1^st^/2^nd^ factor were analogous under the conditions in the young adults at the self-selected and fast walking speeds as well as the elderly at the self-selected walking speeds. Thus the energy flow patterns for those three conditions corresponding to the 1^st^ and 2^nd^ factors were summarized as the following two kinds of representative energy flow characteristics. Notably the significance and sign of the energy flow elements of the elderly at the fast speed were totally opposite to the two representative patterns.

**Table 1:** Correlation coefficient matrix of the eleven energy flow elements.

	Pelvis Distal Flow	Hip Power	Thigh Proximal Flow	Thigh Energy Change Rate	Thigh Distal Flow	Knee Power	Shank Proximal Flow	Shank Energy Change Rate	Shank Distal Flow	Ankle Power	Foot Proximal Flow
Pelvis Distal Flow	**−**	**−**0.764	**−**0.987	**−**0.699	0.970	**−**0.837	**−**0.983	**−**0.987	0.965	0.039	**−**0.952
Hip Power	**−**0.764	**−**	0.858	0.606	**−**0.844	0.329	0.710	0.670	**−**0.732	**−**0.080	0.719
Thigh Proximal Flow	**−**0.987	0.858	**−**	0.708	**−**0.983	0.748	0.960	0.953	**−**0.951	**−**0.051	0.937
Thigh Energy Change Rate	**−**0.699	0.606	0.708	**−**	**−**0.564	0.510	0.580	0.625	**−**0.533	**−**0.022	0.526
Thigh Distal Flow	0.970	**−**0.844	**−**0.983	**−**0.564	**−**	**−**0.741	**−**0.969	**−**0.949	0.971	0.054	**−**0.957
Knee Power	**−**0.837	0.329	0.748	0.510	**−**0.741	**−**	0.883	0.895	**−**0.859	0.230	0.866
Shank Proximal Flow	**−**0.983	0.710	0.960	0.580	**−**0.969	0.883	**−**	0.990	**−**0.993	0.046	0.985
Shank Energy Change Rate	**−**0.987	0.670	0.953	0.625	**−**0.949	0.895	0.990	**−**	**−**0.967	**−**0.038	0.953
Shank Distal Flow	0.965	**−**0.732	**−**0.951	**−**0.533	0.971	**−**0.859	**−**0.993	**−**0.967	**−**	**−**0.118	**−**0.997
Ankle Power	0.039*	**−**0.080*	**−**0.051*	**−**0.022*	0.054*	0.230	0.046*	**−**0.038*	**−**0.118	**−**	0.188
Foot Proximal Flow	**−**0.952	0.719	0.937	0.526	**−**0.957	0.866	0.985	0.953	**−**0.997	0.188	**−**

*Correlation coefficients with poor significance (<0.1).

**Table 2:** Explained variance of extracted 1st and 2nd factors of the energy flow data in the young adults and the elderly.

Factor	Young Adults		Elderly	
	Self-Selected	Fast	Self-Selected	Fast
1^st^ Factor	57%	60%	50%	62%
2^nd^ Factor	36%	35%	46%	33%
**Total**	**93%**	**95%**	**96%**	**95%**

**Table 3:** Loadings of energy flow elements in extracted factors for the young adults and elderly during the swing phase at the self-selected and fast walking speeds.

Energy Flow	Young Adults				Elderly			
	Self-Selected		Fast		Self-Selected		Fast	
	1^st^ Factor	2^nd^ Factor	1^st^ Factor	2^nd^ Factor	1^st^ Factor	2^nd^ Factor	1^st^ Factor	2^nd^ Factor
Pelvis Distal Flow	0.79	−0.61	0.80	−0.59	0.70	−0.72	−0.89*	0.45
Hip Power	−0.30	0.91^**§**^	−0.38	0.90^**§**^	−0.37	0.92^**§**^	0.98^**§**^	−0.13
Thigh Proximal Flow	−0.70	0.71	−0.74	0.68	−0.59	0.81*	0.94*	−0.34
Thigh Energy Change Rate	−0.27	0.76	−0.04	0.89*	0.05	0.90*	0.80*	0.43
Thigh Distal Flow	0.75	−0.63	0.80	−0.59	0.65	−0.75	−0.88*	0.46
Knee Power	−0.95^**§**^	0.13	−0.90*	−0.24	−0.93^**§**^	−0.14	0.001	−0.90^**§**^
Shank Proximal Flow	−0.87*	0.49	−0.90*	0.44	−0.81*	0.59	0.78	−0.62
Shank Energy Change Rate	−0.87*	0.48	−0.85*	0.51	−0.73	0.67	0.89*	−0.45
Shank Distal Flow	0.86*	−0.48	0.93*	−0.37	0.86*	−0.50	−0.65	0.76
Foot Proximal Flow	−0.87*	0.47	−0.93^**§**^	0.33	−0.87*	0.46	0.62	−0.77

*Significant absolute loading (>0.8); ^**§**^Highest loading in each column.

**Figure 3:**
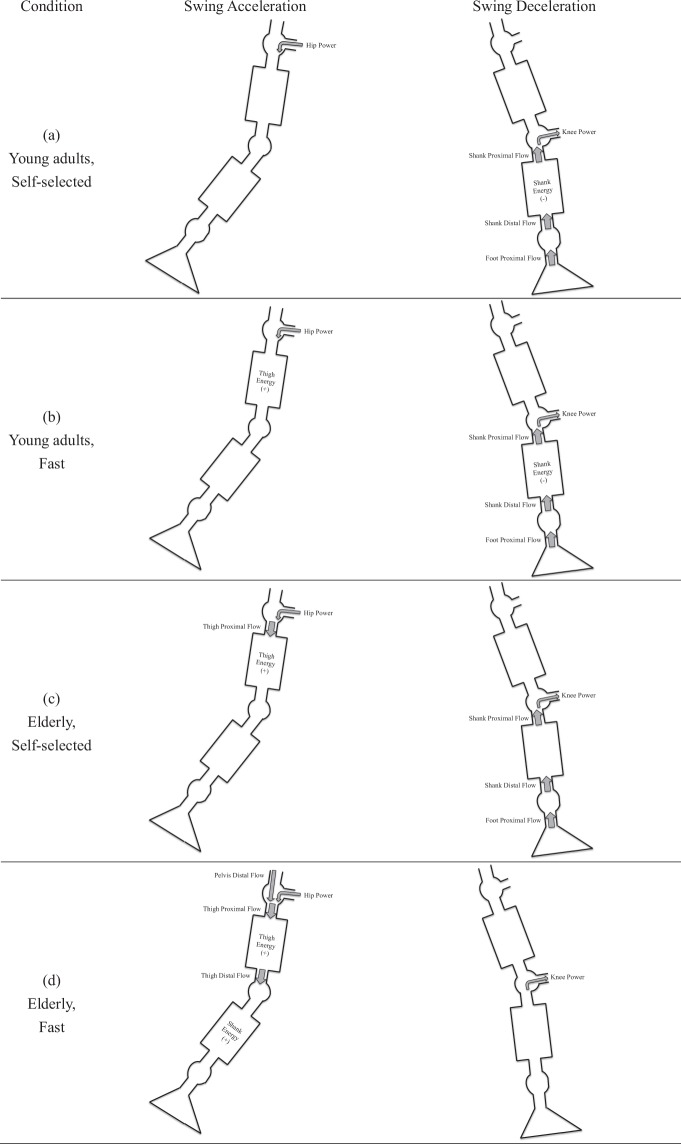
Energy flow patterns corresponding to the swing acceleration and swing deceleration in the young adults and the elderly at the self-selected and fast walking speeds.

The first representative energy flow characteristics could be patterned in terms of the 1^st^ factor, which was with highest loading at the knee power, with most significant loadings below the knee joint, and insignificant hip power and thigh energy change rate (Table 3). This characteristic accordingly showed a knee-dominated pattern. It was noted that the signs of the energy flow elements in this pattern perfectly agreed with the signs of the energy flow profiles during the late stage of the swing phase as shown in Fig. 2. Thus the knee-dominated pattern represents the energy flow characteristic of swing deceleration. It could be illustrated as an upward energy transfer since there was a substantial amount of energy flowing from the foot all the way up to the pelvis and the segmental energy change rates decreased together with the knee power absorption. The second energy flow characteristic could be found in the 2^nd^ factor with highest loading at the hip energy flow and most significant loadings above the knee joint. Since the signs of the energy flow elements of this hip-dominated pattern agreed with the signs of the energy flow profiles during the early stage of the swing, this pattern shows the energy flow characteristic of swing acceleration. It can also be depicted as a downward energy transfer for the segmental energy change rates increased together with the hip power generation (Fig. 3a–c). Following the same process to reveal the energy flow pattern of the elderly during the fast walking, different patterns were observed that the 1^st^ factor oppositely corresponds to swing acceleration with the signs of the loadings agreed with those in the 2^nd^ factor of the representative energy flow, and that the 2^nd^ factor of the elderly during the fast walking corresponds to swing deceleration. In addition, the high-loading energy flow elements in this condition were especially above the knee during the swing acceleration, and there was a centered pattern exclusively at the knee power during the swing deceleration (Fig. 3d).

## Discussion

This study demonstrated a systematic approach to extract the energy flow characteristics of the swing leg in the young adults and elderly. The major findings were: (1) the energy flows of the swing leg showed different patterns between the early swing and the late swing with negligible ankle power; (2) the young adults showed similar energy flow characteristics of the swing leg for both fast and self-selected walking speeds, while the elderly showed an especially opposite energy flow pattern at the fast walking speed; and (3) the hip power and the knee power mainly correspond to the swing acceleration and deceleration, respectively. Our work demonstrated a valuable analytic scheme to explore the changes of the gait characteristics and potentially the mechanisms of the tripping risk in elderly.

Energy flow analysis is a powerful tool to observe the energy transfer through the segment compared to the traditional joint angle or joint moment analysis. In this study, the representative energy flow pattern of the entire lower extremity clearly visualize the difference of the energy transfer between the early phase and late phase of the swing leg. During the early swing phase, the energy source to propel the lower extremity could be composited of the negative pelvis distal flow (i.e. energy flowing out of the distal pelvis) and the positive hip power (i.e. energy generation from the hip), collectively transfer into the thigh and then the shank, with the trivial knee power (Fig. 2a). This downward energy was partially contributed by the thigh and shank with positive thigh energy change rate and shank energy change rate (Fig. 2e), and the shank distal flow eventually become the energy source of the foot due to the insignificant role of the ankle power (Fig. 2i). During the late swing phase, which is usually the deceleration phase of the swing leg, there was a substantial amount of energy that required to be absorbed by the segments from the negative thigh/shank/foot energy change rate as well as by the joint from the negative knee power. Thus the energy flow analysis could be used to reveal the transfer of the mechanical energy across multiple segments and joints of a swing leg.

The energy flow model showed the merit to illustrate the changes of the different sources of energy throughout the lower extremity based on the energy-time plot. Nevertheless, it could only be noted that the elderly at the fast walking speed especially showed the great energy fluctuation and great individual variations upon the comparisons among the different conditions (e.g. the young adults and elderly at the self-selected or the fast walking speed) (Fig. 2). In addition, there’s no clear definition of the time event in the swing phase of the gait for the dedicated comparisons among the conditions. Although researchers/clinicians usually acknowledge that the swing phase consists of the acceleration and deceleration stage, unlike the well-defined time events such as heel strike, heel off, and toe off during the stand phase, the definitions for the initial swing, mid-swing, and terminal swing stage are vague given that the swing limb is off the ground. The high-dimensional and temporal-dependent characteristics of the energy flow elements in different conditions still need a systematic method to be easily compared and comprehended.

The energy flow model utilized in previous studies mostly focused on analyzing an instant gait event^17,18^. The current study applied the factor analysis to reduce the high-dimensional dataset and showed that the hip power and knee power typically correspond to the acceleration and deceleration phase of the leg swing respectively. As a representative pattern in the young subject at the self-selected walking speed, the 1^st^ factor highly covaried with energy flow elements especially below the knee (i.e., the knee power, the shank proximal flow, the shank energy change rate, the shank distal flow, and the foot proximal flow) with mostly being negative which means the energy was expelled from the segment/joint (Table 3). Moreover, this flow pattern was dominated by the knee power absorption among those high-loading parameters. As shown in Fig. 3a, the knee-dominated flow pattern could be visualized as an upward energy transfer, i.e. the energy flows from distal to proximal for absorbing the segmental energy. Accordingly, the 1^st^ factor could be the governing factor for swing deceleration during the late swing. On the other hand, the 2^nd^ factor mainly attributed to the energy flow elements above the knee with positive values which means the energy was gained in the segment/joint. This pattern was dominated by hip power generation with highest loadings. Thus the 2^nd^ factor was a hip-dominated flow pattern with a downward energy transfer from proximal to distal for storing the segmental energy and corresponds to the leg acceleration during the early swing. The current results evidenced the previous conception that the hip power and knee power play roles during the early and late swing phases^10^. The current study further showed that the 1^st^ factor explained more variance than the 2^nd^ factor (Table 2), which could imply that people put more efforts on the deceleration than acceleration.

The identified energy flow patterns based on the factor analysis also help to reveal the difference in the control mechanism of the lower extremity between the young adults and elderly. In young adults at the self-selected and fast walking speeds, the energy flow characteristic was functionally analogous, that is, the knee power dominated the 1^st^ factor to decelerate the swing leg, while the hip power dominated the 2^nd^ factor for leg acceleration. The elderly showed similar characteristics to the young adults at self-selected speed. But their pattern changed fundamentally at the fast walking speed such that the 1^st^ factor oppositely became the governing factor for the leg acceleration since the high-loading energy flow elements were mostly positive and above the knee, and the 2^nd^ factor became the one corresponded to the swing deceleration, i.e. the high-loading energy flow elements were mostly negative and below the knee. Compared to the corresponding energy flow patterns of the young adults, the 1^st^ factor of the elderly during the fast walking showed the overwhelming highest loading at the hip power, and the 2^nd^ factor showed a centered high loading pattern exclusively at the knee power (Table 3). In addition, the 1^st^ factor for swing acceleration in elderly at the fast walking explained even more variance than the 1^st^ factor for swing deceleration in other conditions (Table 2). In other words, during the fast walking, the elderly especially put more efforts on the acceleration than the deceleration. Judge *et al*. reported that the hip flexor power in elderly would increase not only for assisting swing leg advancement but also for compensating the decreased ankle plantarflexor power^12^. De-Vita and Hortobagyi also found that the hip extensor moment and power would increase together with a reduction of ankle and knee power in elderly at the fast walking speed^19^. The enhanced role of the hip to drive the swing could increase the propelling speed of the leg at the cost of augmenting the sway of the center of mass around the lumbar area. Moreover, the great reliance on the knee to decelerate the swing leg and the reduced efforts on the deceleration may increase the difficulty to precisely place the foot. Thus those compensative strategies used by the elderly could be the reasons leading to the tripping or even fall when they perform a challenged walking such as crossing the street, chasing the bus, or doing a brisk walk exercise.

Several study limitations should be addressed. First, only the energy characteristics during the swing phase were discussed. The swing phase is the focus of this study because the unsuccessful advancement of the leg could be critical issues on tripping in the elderly. Our study was the first to systematically reveal to distinguishable difference of the energy flow characteristics of the swing leg in elderly. Second, the energy flow analysis in this study focused on the energy changes of the segments rather than the energy magnitudes. The magnitudes of the energy vary among subjects, but the pattern of the energy changes across the segments would be analogous and could be appropriate for revealing the control strategy of human movements. Finally, the mechanical energy discussed in this study was to highlight the personal ability to drive the limb against the surrounding challenges. Future study was warranted to incorporate with the electromyography or the cardiopulmonary function measures to facilitate the understanding of the comprehensive energy transfer both mechanically and biologically.

In conclusion, the young adults demonstrated similar energy flow characteristics of the swing leg at both the fast and the self-selective walking speeds, in which they put more efforts on the swing deceleration than on the swing acceleration. Nevertheless, the elderly showed a distinct energy flow pattern at the fast walking that they put significantly great hip and thigh efforts on the swing acceleration but exclusively rely on the knee function to decelerate the swing leg, which might lead to an unstable gait. This study had explicitly revealed the energy change processes occurred during the leg advancement, and the systematic analysis of the energy flow characteristics showed the potential to be further extended to different applications such as the pathological gait, the athlete performance, and the robotic-assisted rehabilitation.
